# Cross-Sectional and Longitudinal Evaluation of the Social Network Use Disorder and Internet Gaming Disorder Criteria

**DOI:** 10.3389/fpsyt.2018.00692

**Published:** 2018-12-21

**Authors:** Polyxeni Bouna-Pyrrou, Birte Aufleger, Simona Braun, Manja Gattnar, Sofia Kallmayer, Helena Wagner, Johannes Kornhuber, Christiane Mühle, Bernd Lenz

**Affiliations:** Department of Psychiatry and Psychotherapy, Friedrich-Alexander University Erlangen-Nürnberg (FAU), Erlangen, Germany

**Keywords:** internet addiction, social network use disorder, internet gaming disorder, validity, reliability, long-term evaluation

## Abstract

**Background:** The inclusion of internet gaming disorder (IGD) as a condition warranting more research in the DSM-5 has led to a rapid increase of research on addictive internet activities. Further evaluation of the criteria for IGD and social network disorder (SND) is needed.

**Objective:** To assess the internal consistency, construct validity, retest-reliability, and long-term stability of SND and IGD criteria in German-speaking cohorts.

**Method:** We conducted total and sex-specific analyses on data from two cross-sectional and longitudinal studies, one sample of 192 participants enriched for internet use and another community-based sample of 2316 individuals.

**Results:** First, independent from assessment setting (online, telephone, on-site) and gender, we found acceptable to good internal consistency for SND and IGD criteria (Cronbach's α 0.690–0.774 for SND and 0.743–0.866 for IGD, respectively). Second, positive Spearman correlations between the sum of affirmed criteria and established scales of pathological internet use (ρ 0.395–0.783) and time spent on the social networking sites or internet gaming (ρ 0.317–0.761) confirmed convergent validity. Moreover, the sum of affirmed criteria related positively to attentional impulsivity (ρ_max_ 0.311), urgency (ρ 0.124–0.200), and neuroticism (ρ_max_ 0.210), and negatively to perseverance (ρ −0.245— −0.098) and conscientiousness (ρ_min_ −0.257). Finally, SND and IGD criteria showed high retest stability (SND ρ 0.653–0.826, IGD ρ 0.714–0.825, respectively). However, participants scored higher on SND and IGD scales during the online compared to the on-site assessment. The 2-year follow-up revealed an increase in affirmed SND and IGD criteria.

**Conclusion:** Our data support good psychometric properties of the SND and IGD criteria and outline the addictive potential of social networking sites.

## Introduction

During the last decade, the research field of addictive internet use has expanded exponentially. The prevalence of addictive internet use worldwide varies from 5% in Asia (1, 2) to 1–2% in Europe (3, 4). However, there are numerous methodological problems to overcome. The comparability of available studies is limited by variable diagnostic criteria, assessment tools, and different cohorts investigated for pathological internet use. So far, there is no gold standard with which to measure addictive internet use.

In 2013, the American Psychiatric Association included internet gaming disorder (IGD) in the appendix of the fifth version of the Diagnostic and Statistical Manual for Mental Disorders (DSM-5) as a condition warranting further research (5). The restriction to internet gaming has been criticized for many years, as other potentially addictive activities on the internet are ignored (6, 7). Recently, studies have shown that excessive use of social networks is associated with symptoms typical of substance use disorders (8, 9). Instruments assessing the pathological use of social networks share common components with the diagnostic criteria for IGD (9). Therefore, in a previous investigation, we adapted the nine DSM-5 criteria for IGD to the pathological use of social networks (“SND” for social network use disorder) (10). Further research on the internal consistency, construct validity, retest-reliability, and the long-term stability of these criteria, in particular for SND, is needed.

Impulsivity and personality may provide convergent validity because their role in addictive disorders is well-established. Impulsivity is supposed to be both a cause and a consequence of substance use (11). Impulsivity is described as a vulnerability marker for substance use disorder. In an animal study, Perry et al. showed that rats with higher levels of impulsivity consumed more cocaine than those with non-impulsive behavior (12). Continuous substance use reduces behavioral self-control, probably mainly due to alterations in the prefrontal cortex (13, 14). Brain imaging studies have shown that the dopaminergic striatal-thalamic-orbitofrontal circuit mediates the rewarding effects of cocaine and other substances (15) which are responsible for impulsive drug abuse. Impulsivity varies across the population; a high degree of impulsivity leads to dysfunctional behavior such as substance and non-substance use disorders (16, 17). Impulsivity is one of the most powerful mechanisms in the beginning of addiction (18, 19). In the last years, studies have reported associations between impulsivity and excessive internet use (20, 21). Some authors go even further and suggest that addictive internet use can be classified as an impulse disorder or is at least related to impulse control disorders (22, 23). The model of IGD as an impulse control disorder is supported by the association between IGD and diminished self-regulation. Dong and Potenza postulated in 2014 a cognitive behavioral model for IGD (24). This model consists of three cognitive domains that could lead to internet gaming disorder including motivational impulses related to reward-seeking and stress reduction, behavioral control relating to executive inhibition, and decision-making.

The association between IGD and higher impulsivity is supported by a rapidly increasing number of brain imaging studies (25, 26). Major findings are structural and functional alterations similar to those of substance use disorders. In a recent review of brain imaging studies regarding IGD, Kuss et al. concluded, that affected individuals had difficulties in response-inhibition and emotion regulation as well as in functioning of the prefrontal cortex. Furthermore, subjects showed impairments in decision-making capabilities and in their neuronal reward system (27). In a study by Park et al., individuals with excessive internet gaming showed abnormalities in their resting state activity in the orbito-frontal cortex and striatum as seen previously in impulse control disorders (28). These regions are relevant for impulse control and reward mechanisms. Moreover, increased striatal volume has been found in individuals with IGD (29). In this study, the volume of the nucleus accumbens correlated with the score of the Internet Addiction Test (IAT) and the caudate volume correlated with the Stroop performance task, a tool to evaluate executive functions. Other studies have found decreased connectivity in the amygdala, orbito-frontal cortex and dorso-lateral prefrontal cortex, the anterior cingulate cortex as well as the striatum in participants with higher Internet addiction scores (30). Nevertheless, these recent results of brain imaging studies are limited by the cross-sectional design as well as the lack of sex-specific analyses (31).

The data on pathological use of social networks is even more restricted. There is some evidence that social network use on smartphones relates to higher impulsivity quantified by the Chinese version of Eysenck and Eysenck's impulsiveness scale and by a 20-item inventory that was modified from Young's Internet Addiction Test (32).

Moreover, neuroticism might represent a general health risk factor and predisposes to addiction (33). Accordingly, higher scores on neuroticism have been associated with IGD (34, 35). Individuals with high levels of neuroticism tend to feel anxious, depressed, and guilty. A possible explanation for the association between neuroticism and IGD is that internet gaming reduces loneliness and relieves anxiety. As far as we know, the association between personality and SND has not been studied yet.

It is also important to note, that longitudinal studies regarding the DSM-5 IGD criteria in adults are rare and do even not exist for SND, as far as we know; hence, the stability of these criteria has not been examined properly.

Against this background, the goal of the present study was to evaluate the internal consistency, construct validity, retest-reliability, and long-term stability of the SND and IGD criteria. We determined these parameters in two cross-sectional and longitudinal investigations.

Studies have shown that male sex might be a risk factor for IGD, whereas females are more prone to addictive behavior related to the use of social networks (10, 36–39). Hoeft et al. (40) described a higher activation and functional connectivity in the meso-cortico-limbic system in male compared to female IGD patients. This implicates gender differences regarding the reward system in individuals with IGD. So far, there have only been limited sex-specific analyses; consequently, we analyzed females and males separately.

## Methods

We conducted two longitudinal studies using a series of questionnaires for addictive internet use, impulsivity, and personality. German language was used in both surveys. We collected sociodemographic parameters such as age, marital status, and level of education.

### Study 1: Repeated Online, Telephone, and On-site Assessments

From December 2014 to August 2017, 3077 individuals, who had been recruited through e-mail, social networks, and public postings, completed an online survey and stated actively that they had answered all questions honestly. We called subjects who were willing to potentially participate in an on-site assessment and tried to enrich the sample for individuals affirming more IGD and/or SND criteria. As a result, 266 subjects were interviewed via telephone and 192 individuals of these were additionally tested on-site in the Department of Psychiatry and Psychotherapy of the Friedrich-Alexander University Erlangen-Nürnberg (FAU, Erlangen, Germany). The following median days passed between the different assessments: online to telephone 28 days, telephone to on-site 15 days, online to on-site 46 days.

### Study 2: Large online cohort

In June and July 2016, 14,027 SoSciPanel members (www.soscisurvey.de) were invited to participate in a standardized 20 min online survey presented on the online platform SoSci Survey. Interested parties were motivated by the chance to win Amazon gift cards. The online survey was started by 2638 participants. We used the following exclusion criteria: Premature discontinuation (*n* = 306), did not state having answered honestly or repeated participation (*n* = 13), and a DEGRADE quality score over 200 (indicator for missing answers and too rapid completion (*n* = 3). In total, we analyzed data from 2316 study participants. For further information on the study cohort, see Lenz et al. (41).

*Long-term follow-up:* In 2016, we were able to reassess a subsample of 307 individuals (191 females, 116 males, median age at the present survey 33 years, interquartile range 27–49) within Study 2 who had already participated in our previous online survey conducted in 2013 and 2014 (10) (median days of follow-up 838, interquartile range 835–840).

### Instruments

We used diverse questionnaires to assess internet use, impulsivity, and personality.

#### Internet Use

Regarding IGD, we used the German translation of the DSM-5 criteria (5, 42). Affirmative answers to the nine dichotomous items (yes/ no) were summed to a total score. Analogous to the nine IGD criteria, we designed a German questionnaire for the pathological use of social networks (10). To validate the SND and IGD criteria, we used the following parameters: (i) Compulsive Internet Use Scale (CIUS) (21), a 14-item self-reported scale including loss of control, withdrawal symptoms, coping/mood modification, preoccupation, and conflict; the test is based on the DSM-IV diagnoses for substance dependence and pathological gambling (APA, DSM-IV). (ii) IAT; the IAT is a 20-item self-reported questionnaire that measures the degree of internet addiction based on criteria for pathological gambling and substance dependence (43, 44). (iii) Two scales for pathological use of social networks established by Turel and Serenko, one scale reflecting addiction and one engagement (9); we adapted these scales to the use of internet games and calculated a composite score by summing the scores of the 5 items answered along a 7-point scale. (iv) Self-reported average and maximum time spent weekly on social networking sites or internet games during the previous 12 months. “Maximum” refers to the two weeks in the preceding year during which the participants reported to had used the internet the most.

#### Impulsivity and Personality

We used the Barratt Impulsiveness Scale 15 (BIS-15) (45), a revised and shorter version of BIS-11, to assess impulsivity. It consists of 15 items on a four-point scale (rare/never = 1, occasional = 2, often = 3, almost always/always = 4). Impulsivity is captured by the following subscales: non-planning impulsivity, motor impulsivity, and attentional impulsivity. In the online surveys, participants completed the German impulsivity questionnaire “Skala Impulsives Verhalten (I-8)” (46). Regarding personality factors, we used the 10-item Big Five Inventory (47), a short version of the established Big Five Inventory, which allows assessing the Big Five using only two items per dimension (extraversion, neuroticism, openness to experience, conscientiousness, agreeableness).

### Ethical Approval

This project was approved by the Ethics Committee of the Friedrich-Alexander University Erlangen-Nürnberg (FAU; ID 177_13 B).

### Statistical Analyses

We applied Cronbach's α as an internal consistency estimate of the reliability of the test scores. Correlations were analyzed with Spearman's method. We used Mann-Whitney *U* tests to compare values between independent samples and Wilcoxon signed-rank and Friedman tests to analyze differences between related samples. *P* < 0.05 for two-sided tests was considered to be statistically significant. In pairwise comparisons, Bonferroni adjusted *P*-values are reported. Data were analyzed using IBM SPSS Statistics Version 24 for Windows (SPSS Inc., Chicago, IL, USA) and Graph Pad Prism 5 (Graph Pad Software Inc., San Diego, CA, USA).

## Results

### Sample Description

The two studies did not differ with regard to sex (χ^2^ < 0.1, df = 1, *P* = 0.879). Participants of Study 1 were younger (*U* = 75,076, *P* < 0.001), more often single (χ^2^ = 14.8, df = 1, *P* < 0.001), less often married (χ^2^ = 82.9, df = 1, *P* < 0.001) or divorced (χ^2^ = 17.7, df = 1, *P* < 0.001), and were less likely to live with an underage child (χ^2^ = 42.5, df = 1, *P* < 0.001). Due to our recruitment procedure, subjects of Study 1 reported longer mean and maximum time spent on social networking sites (mean, *U* = 130,305, *P* < 0.001; maximum, *U* = 114,838, *P* < 0.001) and longer mean and maximum time spent on internet games (mean, *U* = 187,229, *P* < 0.001; maximum, *U* = 165,949, *P* < 0.001), and they affirmed more SND and IGD criteria (SND, *U* = 162,167, *P* < 0.001; IGD, *U* = 182,169, *P* < 0.001) (Table 1).

**Table 1:** Sociodemographic characteristics.

	**Study 1**				**Study 2**			
	**Females**		**Males**		**Females**		**Males**	
*N*	99		93		1181		1135	
Age (years)	22 (21–24)		23 (21–27)		32 (25–47)		37 (27–53)	
Single (%)	40.4		52.7		30.7		34.8	
Married (%)	0.0		4.3		30.4		37.4	
Divorced (%)	2.0		0.0		11.2		9.6	
≥1 underage child (%)	1.0		1.1		19.7		20.6	
Sum of school, training, and university years	14 (13–17)		15 (13–18)					
School years					7 (5–8)		7 (5–8)	
Training years					1 (1–4)		2 (1–4)	
University years					8 (1–12)		9 (1–13)	
Past year time spent in social networks^a^								
Mean (h/week)	10 (7–18)		7 (3–15)		4 (1–10)		3 (1–7)	
Max (h/week)	20 (10–30)		14 (5–25)		6 (2–14)		4 (1–14)	
Past year time spent on internet gaming^a^								
Mean (h/week)	0 (0–2)		3 (1–14)		0 (0–2)		0 (0–3)	
Max (h/week)	0 (0–8)		15 (4–40)		0 (0–5)		1 (0–10)	
Number of affirmed criteria^a^ (%)	SND	IGD	SND	IGD	SND	IGD	SND	IGD
0	23.2	72.7	46.2	45.2	48.0	79.0	63.3	74.6
1	21.2	13.1	17.2	19.4	19.8	8.6	18.4	10.6
2	15.2	4.0	12.9	11.8	12.6	5.2	9.1	6.3
3	11.1	2.0	8.6	8.6	8.5	3.1	4.0	3.2
4	14.1	6.1	6.5	5.4	5.5	1.9	2.2	2.8
5	11.1	1.0	2.2	5.4	2.7	0.8	1.4	1.0
6	2.0	1.0	4.3	2.2	0.5	0.8	0.7	0.8
7	1.0	0.0	1.1	1.1	1.1	0.2	0.4	0.5
8	0.0	0.0	1.1	1.1	0.3	0.2	0.3	0.0
9	1.0	0.0	0.0	0.0	0.9	0.3	0.4	0.3

*The table shows absolute and relative frequencies as well as medians with interquartile ranges*.

a *On–site assessment in Study 1 and online assessment in Study 2. Missing values for the categories single, married, divorced, ≥1 underage child, training years, and university years <2%. SND social network use disorder, IGD internet gaming disorder*.

In both studies, females fulfilled more SND criteria (Study 1, *U* = 3385, *P* = 0.001; Study 2, *U* = 548,888, *P* < 0.001) and reported longer mean (Study 1, *U* = 3445, *P* = 0.003; Study 2, *U* = 568,014, *P* < 0.001) and maximum time spent on social networking sites (Study 1, *U* = 3488, *P* = 0.004; Study 2, *U* = 584,453, *P* < 0.001). By contrast, male study subjects fulfilled more IGD criteria (Study 1, *U* = 3253, *P* < 0.001; Study 2, *U* = 640,954, *P* = 0.014) and reported longer mean (Study 1, *U* = 2229, *P* < 0.001; Study 2, *U* = 618,195, *P* < 0.001) and maximum time spent on internet games (Study 1, *U* = 2192, *P* < 0.001; Study 2, *U* = 609,712, *P* < 0.001).

### Internal Consistency of SND and IGD Criteria in Diverse Assessment Settings

The Cronbach's α for the SND criteria ranged between 0.690 and 0.774. For the IGD criteria, we found Cronbach's α values between 0.743 and 0.866. We did not observe distinct differences in internal consistency regarding assessment setting (online, telephone, on-site) and sex (Table 2).

**Table 2:** Cronbach's α depending on assessment site and sex.

		**SND**			**IGD**		
	**Assessment site**	**Total cohort**	**Females**	**Males**	**Total cohort**	**Females**	**Males**
Study 1	Online	0.774	0.766	0.763	0.846	0.866	0.814
	Telephone	0.710	0.690	0.695	0.806	0.851	0.760
	On–site	0.747	0.712	0.774	0.762	0.743	0.755
Study 2	Online	0.749	0.751	0.732	0.779	0.786	0.773

*SND, social network use disorder; IGD, internet gaming disorder. Number of cases females/males; Study 1: online 96/93, telephone 96/88 for SND and 90 for IGD, on–site 99/93; Study 2 1181/1135*.

### Construct Validity

To assess convergent validity, we correlated the SND and IGD criteria with scores of established questionnaires of pathological internet use, times spent on social networking sites or internet gaming, impulsivity measures, and personality factors (Table 3). For both sexes, we found moderate to strong positive correlations of the number of affirmed SND and IGD criteria with the CIUS, the IAT, and the Serenko scale scores as well as with the time spent with social networking sites and internet games. Notably, correlations of affirmed criteria with CIUS and IAT scores were stronger for SND than for IGD and correlations of affirmed criteria with time spent on internet activities and Serenko scale scores were stronger for IGD than for SND. With regard to impulsivity, more affirmed SND and IGD criteria were related to more attentional impulsivity, higher levels of urgency, and lower levels of premeditation and perseverance. Moreover, extraversion correlated negatively with IGD scores and, SND and IGD scores associated positively with neuroticism and negatively with conscientiousness (Table 3).

**Table 3:** Spearman correlations (ρ) between the number of affirmed SND and IGD criteria and internet use, impulsivity, and Big Five personality.

			**SND**			**IGD**		
			**Total**	**Females**	**Males**	**Total**	**Females**	**Males**
**INTERNET USE**								
Study 1	CIUS^a^		0.646^***^	0.738^***^	0.600^***^	0.492^***^	0.445^***^	0.591^***^
	IAT^b^		0.483^***^	0.534^***^	0.480^***^	0.395^***^	0.395^***^	0.405^***^
	Time spent^a^^,^ ^c^	Average	0.466^***^	0.317^**^	0.514^***^	0.715^***^	0.728^***^	0.616^***^
		Maximum	0.516^***^	0.441^***^	0.505^***^	0.735^***^	0.761^***^	0.642^***^
Study 2	Serenko scales^b^^,^ ^c^		0.660^***^	0.686^***^	0.615^***^	0.755^***^	0.724^***^	0.783^***^
	Time spent^b^^,^ ^c^	Average	0.486^***^	0.492^***^	0.454^***^	0.627^***^	0.609^***^	0.642^***^
		Maximum	0.495^***^	0.505^***^	0.465^***^	0.651^***^	0.627^***^	0.671^***^
**IMPULSIVITY**								
Study 1	BIS-15^a^	Non–planning impulsivity	−0.143^*^	−0.131	−0.049	0.050	−0.126	0.136
		Motor impulsivity	0.154^*^	0.181	0.142	−0.003	−0.122	0.090
		Attentional impulsivity	0.171^*^	0.261^**^	0.160	0.212^**^	0.056	0.311^**^
		Sum score	0.074	0.124	0.124	0.111	−0.110	0.261^*^
Study 2	I-8^b^	Urgency	0.179^***^	0.200^***^	0.124^***^	0.126^***^	0.126^***^	0.140^***^
		Premeditation	−0.067^**^	−0.053	−0.030	−0.049^*^	−0.049	−0.065^*^
		Perseverance	−0.149^***^	−0.217^***^	−0.098^***^	−0.194^***^	−0.139^***^	−0.245^***^
		Sensation seeking	0.009	0.049	0.015	−0.030	−0.037	−0.037
**BIG FIVE PERSONALITY**								
Study 1	BFI-10^b^	Extraversion	−0.009	−0.080	−0.008	−0.173^*^	−0.262^**^	−0.027
		Neuroticism	0.181^*^	0.056	0.210^*^	0.025	0.118	0.112
		Openness to experience	0.135	0.071	0.166	0.017	−0.067	0.122
		Conscientiousness	0.105	−0.002	0.099	−0.257^***^	−0.172	−0.211^*^
		Agreeableness	0.097	0.146	−0.046	−0.175^*^	0.099	−0.311^**^
Study 2	BFI-10^b^	Extraversion	0.050^*^	0.008	0.047	−0.105^***^	−0.089^**^	−0.113^***^
		Neuroticism	0.159^***^	0.126^***^	0.113^***^	0.069^***^	0.086^**^	0.080^**^
		Openness to experience	0.057^**^	0.029	0.054	−0.026	0.019	−0.063^*^
		Conscientiousness	−0.136^***^	−0.192^***^	−0.116^***^	−0.222^***^	−0.189^***^	−0.245^***^
		Agreeableness	−0.009	−0.035	0.008	−0.035	−0.032	−0.036

*The table shows Spearman correlations (ρ) between the number of affirmed SND and IGD criteria (assessed on-site for Study 1 and online for Study 2) and internet use, impulsivity, and personality. SND, social network use disorder; IGD, internet gaming disorder; CIUS, Compulsive Internet Use Scale; IAT, Internet Addiction Test; BIS−15, Barrat Impulsiveness Scale – short version; I−8, Scale Impulsive Behavior−8; BFI−10, Big–Five–Inventory−10*.

a *On–site assessment*,

b *online assessment*.

c *Time spent on social networking sites and Serenko scales adapted to social network use were correlated with SND criteria, and time spent on internet gaming and Serenko scales adapted to internet gaming were correlated with IGD criteria*.

* *P < 0.05*,

** *P < 0.01*,

*** *P < 0.001. Number of cases females/males; Study 1: CIUS 94/89, IAT 96/93, times spent 99/93, BIS−15 99/93, BFI−10 96/93; Study 2: 1181/1135*.

### Retest Reliability

We correlated intra-individual SND and IGD scores from the online, telephone, and on-site assessments with each other and found high retest stability for all combinations in both males and females (Table 4).

**Table 4:** Spearman correlations (ρ) between different assessment sites.

	**Total cohort**		**Females**		**Males**	
	**Online**	**Telephone**	**Online**	**Telephone**	**Online**	**Telephone**
**SND**						
On–site	0.711	0.797	0.696	0.826	0.653	0.745
Telephone	0.744		0.737		0.673	
**IGD**						
On–site	0.766	0.785	0.745	0.714	0.737	0.816
Telephone	0.825		0.809		0.787	

*SND, social network use disorder; IGD, internet gaming disorder. P < 0.001 in all shown correlations. Number of cases females/males; online – on–site SND and IGD 96/93, online – telephone SND 94/88 IGD 94/90, telephone – on–site SND 96/88 IGD 96/90*.

**Figure 1:**
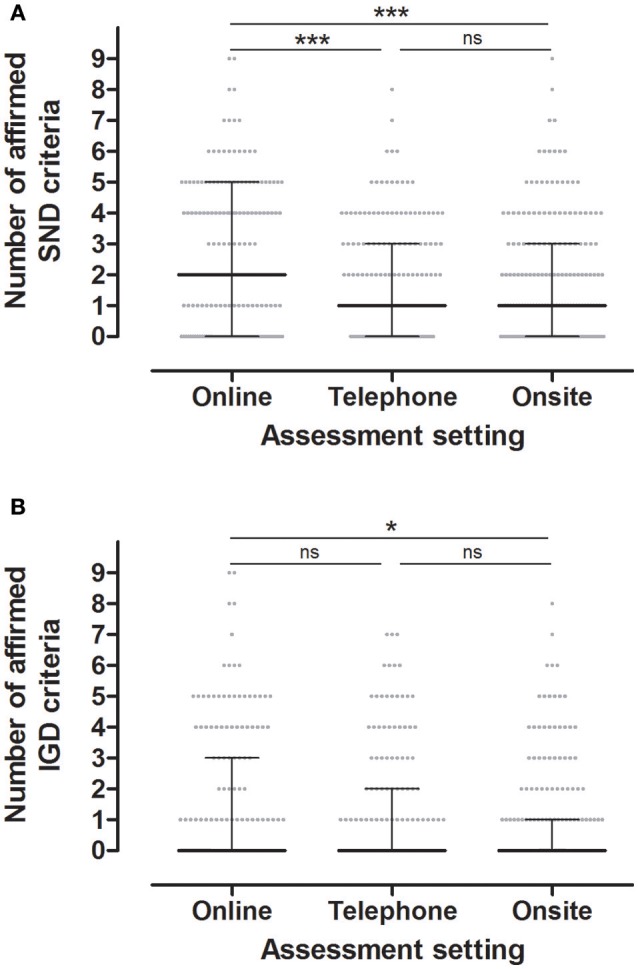
The graphs show median and interquartile range of number of affirmed SND **(A)** and IGD **(B)** criteria in different assessment settings. SND social network use disorder, IGD internet gaming disorder. ^*^P < 0.05, ^***^P < 0.001; ns, not significant.

We tested whether the number of affirmed criteria depended on the assessment setting and found higher scores in the online assessment than in the on-site assessments (Figure 1. SND: Friedman test; total cohort, *n* = 182, χ^2^ = 56.6, df = 2, *P* < 0.001, pairwise comparisons, online vs. telephone *P* < 0.001, online vs. on-site *P* < 0.001, telephone vs. on-site *P* = 0.597; females, *n* = 94, χ^2^ = 26.0, df = 2, *P* < 0.001, pairwise comparisons, online vs. telephone *P* < 0.001, online vs. on-site *P* = 0.004, telephone vs. on-site *P* = 1.000; males, *n* = 88, χ^2^ = 31.2, df = 2, *P* < 0.001, pairwise comparisons, online vs. telephone *P* < 0.001, online vs. on-site *P* = 0.008, telephone vs. on-site *P* = 0.683. IGD: Friedman test; total cohort, *n* = 184, χ^2^ = 22.5, df = 2, *P* < 0.001, pairwise comparisons, online vs. telephone *P* = 0.098, online vs. on-site *P* = 0.016, telephone vs. on-site *P* = 1.000; females, *n* = 94, χ^2^ = 1.1, df = 2, *P* = 0.590; males, *n* = 90, χ^2^ = 25.8, df = 2, *P* < 0.001, pairwise comparisons, online vs. telephone *P* = 0.027, online vs. on-site *P* = 0.001, telephone vs. on-site *P* = 1.000).

### Long-term Follow-up

**Figure 2:**
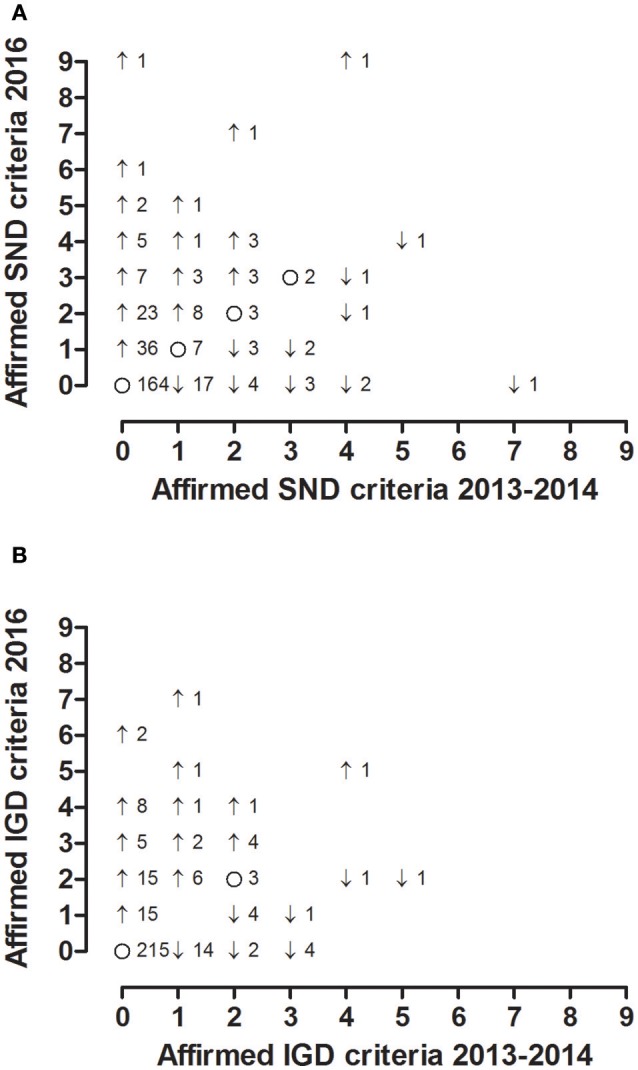
The graphs plot the number of affirmed SND **(A)** and IGD **(B)** criteria quantified during the first survey conducted in 2013 and 2014 (x-axis) and during the second survey conducted in 2016 (y-axis). The figures next to the dots represent the number of underlying individuals. ↑ increase, ↓ decrease, and o no change of affirmed criteria during the follow-up. SND social network use disorder, IGD internet gaming disorder.

Our analyses showed a positive correlation between the number of affirmed criteria during the first survey and the values quantified during the second survey (Spearman correlations; total cohort, *n* = 307, SND, ρ = 0.285, *P* < 0.001, IGD, ρ = 0.355, *P* < 0.001; females, *n* = 191, ρ = 0.290, *P* < 0.001, IGD, ρ = 0.325, *P* < 0.001; males, *n* = 116, SND, ρ = 0.277, *P* = 0.003, IGD, ρ = 0.400, *P* < 0.001). Moreover, we found an increase in the affirmed criteria for IGD and SND both for females and males during the 2-year observation period (increase in affirmed SND/IGD criteria; total cohort, median 0/0, 75% percentile 0/0, 90% percentile 1/0, 95% percentile 1/1; females, median 0/0, 75% percentile 0/0, 90% percentile 1/0, 95% percentile 1/1; males, median 0/0, 75% percentile 0/0, 90% percentile 1/0, 95% percentile 1/1; Figure 2; Wilcoxon signed-rank tests; total cohort, *n* = 307, SND, z = −5.2, *P* < 0.001, IGD, z = −4.2, *P* < 0.001; females, *n* = 191, SND, z = −4.5, *P* < 0.001, IGD, z = −3.2, *P* = 0.001; males, *n* = 116, SND, z = −2.8, *P* = 0.005, IGD, z = −2.8, *P* = 0.006).

## Discussion

The inclusion of the IGD in the DSM-5 has prompted the empirical research of addictive internet use during the last decade by delivering a standardized terminology regarding the pathological use of internet games. In spite of the continuously increasing numbers of studies regarding pathological internet use, classification is still a major limitation for this research, as different, non-standardized measurement tools are being used. We focused on use of social networking sites and internet gaming because these activities exhibit high addictive potential (6) and we aimed at providing evidence for internal consistency, construct validity, retest reliability, and longitudinal stability for the SND and IGD criteria.

In different settings, we found acceptable to good internal consistency for the SND and IGD scales, similar to a former online study (10). The SND and IGD criteria correlated positively with the CIUS scores, IAT scores, Serenko scale scores, and time spent with the respective internet activities. These observations fit the expected pattern and thus contribute evidence of construct validity.

Although impulsivity and neuroticism are established risk factors for IGD, there is a lack of data regarding the addictive use of social networks. Therefore, we investigated the associations of impulsivity and the Big Five model of personality traits with the addictive use of social networks (and internet games). Regarding impulsivity, more affirmed SND and IGD criteria were related to more attentional impulsivity, higher levels of urgency, and lower levels of premeditation and perseverance. Concerning the Big Five model of personality traits, extraversion correlated negatively with IGD scores. Furthermore, SND and IGD scores were associated positively with neuroticism and negatively with conscientiousness. The correlation between neuroticism and the pathological use of internet games is in line with several studies examining IGD and personality factors (48, 49). Mueller et al. even consider neuroticism to be a general risk factor, as it also predicts substance use (34). In the same study, IGD patients showed less conscientiousness, which is also in line with our results.

To our knowledge, this study is the first to investigate whether the assessment setting influences responses to the SND and IGD criteria and their internal consistency. Notably, we detected no systematic effects on internal consistency and conclude that the criteria are well-applicable to different settings (i.e., online, telephone, and on-site assessments) in both sexes. However, participants scored higher on SND and IGD scales during the online compared to the on-site assessment, suggesting that participants downplay the extent of their pathological internet use when in direct contact.

Many studies have shown that males are more prone to the addictive use of video games, whereas females are more often burdened by addictive use of social media (50). In our study, female participants also fulfilled more SND criteria and male participants more IGD criteria.

The need for more longitudinal studies has been emphasized by many authors (51, 52). The available data on the stability of IGD are inconsistent. Some longitudinal studies have shown the high persistence of IGD after 2 years (53), while others report only a low persistence in this time period (54). One survey by Gentile et al. (53) showed that 84% of children with pathological gaming still fulfilled the criteria at the follow-up investigation after 2 years. Moreover, most of the available longitudinal studies focus on risk and protective factors for IGD and do not analyze the stability of the SND criteria. In our Study 2, we found a positive correlation between the number of affirmed criteria at baseline and at the 2-year follow-up for both the SND and IGD criteria. This highlights the long-term stability of addictive social networking and internet gaming. Moreover, we found an intra-individual increase in affirmed criteria during the 2-year observation period which is in line with the globally increasing digitalization.

The vast majority of studies in the internet addiction field are cross-sectional and only a few longitudinal studies concerning adults are available. The strengths of this study include the follow-up investigation. As a higher prevalence of IGD has been reported in males, many previous studies have focused on male individuals. Therefore, we consider our sex-specific analyses as another considerable asset of our study. Moreover, we selected anonymous online questionnaires to minimize confounding by social stigma. However, the self-report method used in this study might also have induced inaccuracy.

As most of the participants in both studies were highly educated, the sample is not entirely representative of the general population. Few subjects fulfilled the DSM-5 cut-off of at least five affirmed IGD criteria. Another limitation is that the sample of Study 1 consisted mainly of students. However, current evidence has shown that students are more vulnerable to developing an addictive use of the internet (55). A further limitation of our study was the recruitment through e-mail, internet advertisements, and social networking sites, which could lead to a selection bias toward internet users. We conducted a number of different analyses and most have not been corrected for multiple testing. Finally, due to the cross-sectional study design, the causality and direction remain to be studied in further detail.

In summary, here we provide multilevel evidence for the good internal consistency, construct validity, retest reliability, and long-term stability of the DSM-5 research criteria for IGD and the adapted criteria for SND. Moreover, our study outlines the addictive potential of social network use.

## Author Contributions

PB-P, CM, and BL conceived and designed the experiments. PB-P, BA, SB, MG, SK, HW, CM, and BL performed the experiments. PB-P, CM, and BL analyzed the data and wrote the paper. JK, BA, and SB commented on the manuscript and provided intellectual input. All authors gave final approvement of the version to be published.

### Conflict of Interest Statement

The authors declare that the research was conducted in the absence of any commercial or financial relationships that could be construed as a potential conflict of interest.
